# Belief in Protecting Others and Social Perceptions of Face Mask Wearing Were Associated With Frequent Mask Use in the Early Stages of the COVID Pandemic in the UK

**DOI:** 10.3389/fpsyg.2021.680552

**Published:** 2021-10-22

**Authors:** Emma Warnock-Parkes, Graham R. Thew, David M. Clark

**Affiliations:** ^1^Department of Experimental Psychology, University of Oxford, Oxford, United Kingdom; ^2^King’s College London, London, United Kingdom

**Keywords:** COVID – 19, face-mask, social fear, cognition, beliefs, social perceptions

## Abstract

Face masks are now seen as a key tool in the world’s recovery from the COVID-19 pandemic. However, during the early stages of the outbreak, face mask use in the United Kingdom (UK) was significantly lower than that of countries equally impacted by the virus. We were interested to explore whether social cognitions played a role in levels of mask wearing. A cross-sectional online survey of UK adults (*n*=908) was conducted in July 2020. Estimated face mask use and thoughts about wearing face masks were assessed using measures developed for this study. Participants also answered questions about their general mood, social anxiety and basic demographic data. Multiple regression was used to examine factors associated with mask wearing. Participants’ estimated mask wearing was low when in public spaces, such as the park (17%) or walking on the high street (36%). However, broadly fitting with UK guidance at the time, rates were considerably higher when in situations of closer proximity to others, such as on public transport (94%), in a shop or café (62%), when speaking to somebody in an enclosed public space (67%) or in a busy area when social distancing was not possible (79%). When looking at estimated mask wearing when in proximity to others, positive social cognitions (e.g., I’ll look confident and competent wearing a mask) were associated with more wearing, whereas negative social cognitions (e.g., I’ll look anxious, I’ll look foolish) were associated with less wearing. These results remained after controlling for factors that have indicated increased risk from COVID-19 (age, gender, ethnicity, presence of a health condition or pregnancy), belief about the health benefit for others and levels of depression and social anxiety. The largest predictors of mask wearing were the amount of people believed wearing a mask would keep others safe and the presence of an underlying health condition. The study findings indicate that future public health campaigns would benefit from a focus on strengthening beliefs about the protective benefits of masks, but also promoting positive social messages about wearing in public (e.g., mask wearing means you are confident and competent).

## Introduction

Face masks have been used worldwide to help reduce the spread of COVID-19. However, prior to the pandemic, they were not a part of UK culture and were rarely seen outside of surgical settings. In March 2020, UK residents were told that there was ‘*little evidence of widespread benefit for members of the public’* (Taken from Feng et al., 2020). However, by July 2020, health campaigns across the UK encouraged mask wearing when in proximity to others. Uptake of mask wearing in the early stages of the pandemic in the UK was seemingly slow. A survey carried out by Imperial College London and YouGov in May 2020 found that only 13% of people in the UK stated that they always wore a face mask when outside the home, compared to 88% of people in Italy and 63% in Spain (Imperial College London, 2020; You Gov/Imperial College London, 2020). Given the potential importance of face mask use in managing COVID-19 and other future pandemics, understanding factors related to poor uptake is fundamental.

The health belief model is one of the most widely used models of social cognition in understanding health-related behaviours (Becker, 1974; Rosenstock, 1974). The model proposes that both threat perception and behavioural evaluation impact on behaviour. Threat perception includes perceived susceptibility to illness and anticipated severity, and behavioural evaluation includes beliefs about the benefits and barriers of a behaviour. Sim et al. (2014) conducted a literature review (pre-COVID-19 pandemic) in the context of the health belief model on the use of face masks as a preventative measure in the community. They found that perceived susceptibility to illness was the most significant factor related to mask wearing. However, they also highlighted the role of perceived benefits and barriers to mask wearing, including personal discomfort and embarrassment. The potential social barrier of embarrassment is interesting and under-researched. Wearing a face mask, unlike other health behaviours (such as attending screening appointments and hand washing), is unique in that the personal choice to wear one or not is constantly visible to others and can have an impact on social communication. Therefore, the fear of social judgement when wearing a mask could have a particular relevance in understanding mask wearing behaviour.

The YouGov survey of mask wearing rates in the UK carried out in May 2020 found that non-mask wearers were more likely to rate themselves as feeling self-conscious and worried about being judged negatively compared to mask wearers (You Gov/Imperial College London, 2020). This indicates a potential role of social embarrassment in predicting low face mask use. However, the survey did not explore the social fears of respondents. The Royal Society (2020) on face masks during the COVID-19 pandemic reviewed literature on adherence with mask wearing and highlighted the importance of socio-behavioural factors but found no studies to date exploring social fears when wearing masks.

There are a limited number of studies on face mask use, but some do mention social embarrassment and fear of social judgement. In an interview study of 137 older adults in Hong Kong about mask wearing to reduce risk of infection from the influenza A/H1N1 pandemic, Zhang and colleagues (Zhang et al., 2019) identified four beliefs related to face mask wearing. These included the following: the perceived susceptibility to and seriousness of the pandemic, modifying factors (e.g., social responsibility to limit spread), cues to action (e.g., seeing other people wearing face masks) and perceived benefits and barriers (such as protection from illness and difficulties breathing). The main barriers to mask wearing during periods of lower risk were difficulty breathing, inconvenience and the image of not looking good socially when wearing a mask. Furthermore, a small interview study of Australian students in 2012 found that participants believed mask wearing would cause them embarrassment and social stigma, with one participant quoted as saying *‘people would look at you weird here’* (Seale et al., 2012). A more recent survey study on the use of masks during COVID-19 in Japan (Nakayachi et al., 2020) found that conforming to societal norms was a bigger predictor of mask wearing than beliefs about the health benefits. This led the authors to conclude that social motivations should be taken into consideration by policy makers when devising public health strategy.

We know of no further research to date that has directly explored social perceptions associated with mask wearing during the COVID-19 pandemic and controlled for the potential influence of social anxiety or depression on cognitions. This is potentially important given we might expect that mood could have an influence on people’s reported cognitions. As mask wearing is likely to be a universal intervention in managing the virus, understanding the factors that predict it will be important. We were interested in whether positive and negative social cognitions about mask wearing are associated with levels of wearing in a UK sample when controlling for a range of other factors.

## Materials and Methods

### Design and Participants

This cross-sectional survey on thoughts about wearing face masks during the COVID-19 pandemic was completed between 07 and 24 July 2020 by 908 participants in the United Kingdom recruited on social media. It should be noted that at the time of administering the survey, guidance in different parts of the UK on face masks/coverings differed, but for the most part, the recommendation was to wear a mask in enclosed public spaces and when in contact with those outside of your own household. In England and Scotland, mask wearing was mandatory on public transport. Some new guidance was announced during the 17 days the survey was open; a Supplementary Table summarises the guidance across different parts of the UK at this time.

Of the participants who gave their age (*n*=883), the average age of participants was 39.15 (SD=13.8), ranging from 18 to 78. Of the participants who gave their gender (*n*=886), the majority identified as female, 68.23% (603), with nearly a third identifying as male 30.46% (269), 0.68% (6) as non-binary and 5 people stated ‘other’. Three participants preferred not to state a gender, and 22 people did not complete this field.

Of the 885 people who responded regarding their ethnicity, 83.95% (743) described themselves as White British or White Other, 4.29% (38) as Mixed ethnicity, 2.26% (20) as Black/Black British, Black African/Caribbean and 6.23% (*n*=55) as Asian/Asian British. A further 1.9% (17) preferred to describe their ethnicity differently, with an additional 12 participants stating they preferred not to say.

### Measures

#### Estimated Percentages of Mask Wearing Scale

Participants were asked to rate how much of the time (0–100%) they would wear a mask in six social situations: (1) walking in the park (2) walking on the high street (3) when on public transport (4) in a shop, café or restaurant (5) when in a busy place and social distancing is not possible and (6) when speaking to somebody in an enclosed public space. For the main analysis, an average of estimated mask wearing in areas of close social proximity was used (including, when on public transport, in a shop, café or restaurant, when in a busy place and social distancing is not possible and when speaking to somebody in an enclosed public space). Participants were also asked to estimate how many people in their local community they thought wore a mask in public.

#### Social Cognitions About Face Masks Questionnaire

To assess fears of social perceptions of mask wearing, participants were asked to consider how much they would believe a range of statements on a 0–100% scale when wearing a mask in a social situation. This included 15 items in total. One item looking at how much participants thought they were keeping others safe when wearing a mask and then two scales:

(1) Negative Social cognitions scale. This consisted of 12 items: People will not understand me; I’ll look: foolish, stupid, awkward, uncool, anxious, sweaty, red; People will: judge me negatively, stare at me, think I’m boring, think I’m rude if I speak with it on.
(2) Positive Social cognitions scale. This consisted of 2 items: I’ll look competent and I’ll look confident

The negative cognitions scale had good internal consistency (Cronbach’s alpha=0.88). The positive cognitions scale had acceptable internal consistency (Cronbach’s alpha=0.73). The SCFM is included in Appendix 1.

#### Ratings of Mood, Anxiety and Self-Consciousness

Participants were also asked to rate how much they believed they would feel anxious and self-conscious when wearing a mask (0–100%).

#### The Patient Health Questionnaire-2

The PHQ-2 is a two-item measure of depression which shows good construct and criterion validity and a sensitivity of 83% and a specificity of 92% for detecting depression (Kroenke et al., 2003).

#### Mini Social Phobia Inventory

The Mini SPIN is a three-item measure of social anxiety which has been found to possess 90% accuracy in detecting the presence of social phobia. It has good reported internal consistency and reliability (Connor et al., 2001).

#### Demographics Questionnaire

Participants were also asked for their age, gender, ethnicity and whether they had an underlying health condition or were pregnant, which could put them at greater risk from COVID-19.

### Procedure

Adults who were UK residents responded to advertisements initially shared on social media by the lead researcher and subsequently reshared by multiple contacts (Facebook and Twitter) and King’s College London research circular. Participants who clicked on the link were taken to an information sheet and asked to give their consent to take part once they had read and fully understood this. If they did not consent, they were withdrawn from the study. All responses were anonymous. Participants were first asked to complete the estimated percentages of mask wearing scale, followed by the cognitions about mask wearing questions and scales, then the Mini-SPIN and PHQ-2 and finally their demographic data.

The study was approved by the relevant ethical committee (MRA-19/20–19,878). Analyses were performed in SPSS version 27.

## Results

### Levels of Face Mask Wearing

**Figure 1:**
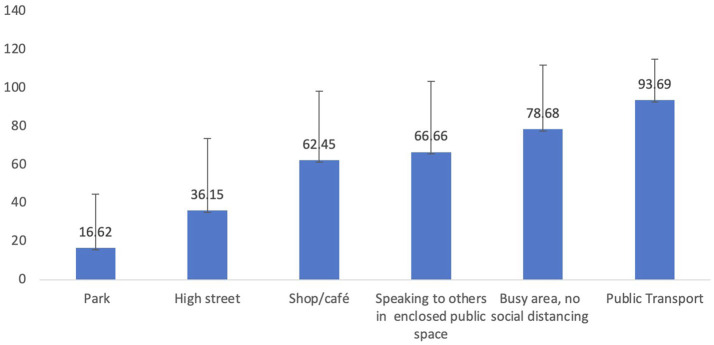
Bar chart showing % estimated mask wearing in a range of situations and standard deviations.

Figure 1 shows the percentages of time that people estimated they wore masks in different social situations. Rates varied considerably depending on the social situation, from an average of 17% when walking in the park up to 94% when on public transport.

Participants were also asked how many people in their local community wear a mask (29.19%, SD=18.07) and how likely they would be to ask somebody to put on a mask if they thought they should be wearing one (24.63%, SD=29.81).

### Mood and Social Anxiety Measures

The mean score on the PHQ-2 was 1.53 (1.64). The authors indicate that a score of 3 is the optimal cut-off when using the PHQ-2 to screen for depression (Kroenke et al., 2003). The mean score on the Mini-SPIN was 2.71 (2.82). A score of 6 or above can discriminate individuals with social anxiety disorder (Seeley-Wait et al., 2009).

### Cognitions and Anxiety About Mask Wearing

Mean scores for ratings of anxiety, self-consciousness and positive and negative face mask cognitions scales are presented in Table 1.

**Table 1:** Mean and standard deviations for anxiety, self-consciousness and cognitions about protecting others and positive and negative social cognitions about mask wearing.

Scale	Mean 0–100% ratings (SD)
How anxious are you wearing FM?	18.24 (28.57)
How self-conscious are you wearing FM?	31.74 (33.51)
I’m keeping people safe	78.62 (30.59)
Negative FM cognitions scale	15.56 (16.53)
Positive FM cognitions scale	38.79 (28.11)

### Factors Associated With Mask Wearing

For the main analysis, a hierarchical multiple regression was conducted with mean estimated mask wearing in enclosed spaces as the dependent variable (computed as the mean of the ratings given for being on public transport, in a shop, café or restaurant, in busy areas when social distancing is not possible, and when speaking to others in an enclosed public space). Variables that were known at the time to increase risk from COVID-19, namely, age, gender (for analysis only male or female was used), ethnicity (for analysis collapsed into white or other ethnic category) and having health complications or pregnancy (for analysis coded as having a underlying health issue or not), as well as symptoms of depression (PHQ-2) and social anxiety (Mini-SPIN) which may influence people’s overall belief ratings, were entered first as covariates. The three independent variables of interest taken from the Social Cognitions about Face Masks Questionnaire (SCFM) were then entered in the second step. These were as follows: the belief that face masks help protect others; the negative social cognitions scale; and the positive social cognitions scale. Statistical assumptions were checked and met for this analysis, and multicollinearity between independent variables was not observed. Results are presented in Table 2.

**Table 2:** Linear regression analyses to determine the influence of each variable on estimated face mask wearing.

	B	SE B	Standardised β	p
Step 1				
Age	0.08	0.07	0.04	0.250
Ethnicity	0.28	2.62	0.00	0.916
Health condition	8.47	2.76	0.11	0.002
Gender	−0.48	2.05	0.01	0.815
PHQ-2	−0.26	0.66	−0.02	0.691
Mini SPIN	0.37	0.38	0.04	0.337
Step 2				
Age	2.15	0.05	0.11	0.000
Ethnicity	1.39	1.81	0.019	0.444
Health condition	4.88	1.91	0.063	0.011
Gender	−0.36	1.41	−0.006	0.799
PHQ-2	0.37	0.45	0.023	0.415
Mini SPIN	0.41	0.28	0.04	0.144
Belief protecting others	0.56	0.024	0.64	0.000
Negative social cognitions	−0.15	0.044	−0.092	0.001
Positive social cognitions	0.11	0.023	0.13	0.000

*R^2^ =0.020 for step 1; ΔR^2^ =0.52 for step 2.*

Greater belief that masks protect others from COVID-19 was the strongest predictor of mask wearing, once controlling for age, gender, ethnicity, having an underlying health condition or pregnancy and mood and social anxiety. Both negative and positive social cognitions were also significant unique predictors. The only demographic factors that predicted mask wearing were having an underlying health condition and age. The cognitions variables, including both health beliefs and social factors, explained an additional 52% (R square change) of the variance in mask wearing, after controlling for the variables entered in the first step.

Participants were asked whether they had any additional comments regarding any concerns about wearing a face mask. Looking at people who reported high scores (over one standard deviation above the mean) on the negative cognitions scale, some participants added additional comments. Some comments illustrate fears of being judged negatively for wearing a mask, for example *‘Not many people wear them where I live and so I do think I will stand out and might look as if I’m taking it too seriously’.* Some respondents commented on the physical discomfort they experience wearing a mask, for example ‘*I will feel uncomfortable and possibly claustrophobic’*, ‘*I cannot breathe’.* Some comments questioned the validity of the virus (e.g., ‘*There’s no virus’)* or the scientific basis of wearing a mask (e.g., *‘They do not work, no point in them’)* and some made comments about restrictions of their liberties (e.g., ‘*It feels oppressive, as though our freedoms are slowly getting eroded, and we are meant to be ok with it’).*

## Discussion

The wearing of face masks is seen as a part of the world’s recovery from the devastating impact of the COVID-19 pandemic. However, rates of mask wearing have varied considerably worldwide and have been notably low in the UK, which, at the time of writing, has suffered one of the largest death rates from COVID-19. In this novel study, we investigated the relationship between social cognitions and face mask wearing in a survey carried out over a short period in July 2020. Respondents reported very high levels of mask wearing on public transport (94%) but much lower rates in wearing in more open spaces in public, such as walking in the park (17%) or down the high street (36%). The results reflect findings by the Imperial College London and YouGov survey carried out in May 2020, that rates of mask wearing outside of the home reported by UK participants was low compared to some other countries equally impacted by the pandemic (such as Italy/Spain).

In line with the health belief model (Becker, 1974; Rosenstock, 1974), beliefs about the health benefits of face masks played a key role in predicting mask wearing, with the belief that wearing a mask keeps others safe from COVID-19 being the biggest predictor. Positive and negative social cognitions about wearing masks were also significant predictors. The results indicate that it is not solely health-related beliefs that contribute to mask wearing and thoughts about how participants came across when wearing one (e.g., looking confident, looking anxious, looking awkward) also predicted mask use. This lends support to the preliminary findings of some other studies carried out during the pandemic that non-mask wearers may worry more about being judged negatively (You Gov/Imperial College London, 2020) and that social motivators also play a role in addition to belief about health benefits (Nakayachi et al., 2020). The findings also support other studies that pre-date COVID-19 that found embarrassment and thoughts about how you look when wearing a face mask may play a role in wearing one (Seale et al., 2012; Zhang et al., 2019).

Given negative social cognitions were a predictor of mask wearing, it may seem surprising that level of depression and particularly social anxiety was not also predictors. In social anxiety, people tend to have negative thoughts about things they say or do socially and that they will appear anxious to others. Therefore, we might expect people higher in social anxiety to worry more about what others would think of them when wearing a face mask. However, common concerns in social anxiety, such as *‘people will see me sweat or blush and think I look anxious’,* may actually be less likely to be activated when wearing a face mask because either the person might think these signs are less visible behind a mask or be more likely to assume that others would interpret blushing or sweating under a mask to be a sign of being hot, rather than being anxious. On the SCFM, respondents who rated items such as ‘I will look anxious (when wearing a mask)’ might have been thinking more about looking anxious about catching COVID-19, than looking socially anxious. A future qualitative study exploring the fears about mask wearing in more depth would be useful.

A strength of the current study is that it was timed just prior to significant changes in legislation in the UK to mandate the wearing of face masks. However, the study is limited as it was a convenience sample, predominantly shared on social media by the research team, potentially leading to bias that limits representation and generalisability. There is also a possible bias in the sample, with more people likely to respond when they had particular views on wearing face masks. Although we tried to keep the survey open for a short period of time to limit the number of legal guidance changes during the period of the survey, there were some changes to the law in Scotland. The majority of the sample is also female.

Face coverings are likely to be an integral part of slowing the COVID-19 pandemic and reducing future lockdowns. The results of this study indicate that during this time, and in future pandemics, clear and consistent messages and policies about mask wearing would be helpful. Furthermore, public health campaigns need to address both health and social beliefs about wearing a mask. Indeed, van der Westhuizen et al. (2020) discuss the importance of shifting the narrative on wearing masks during COVID-19 from being a sign of weakness, as had been depicted by some public figures at the time, to a positive message of wearers as ‘protectors’. Health campaigns presenting wearers as confidently and competently protecting others are likely to be important in improving adherence.

## Data Availability Statement

The raw data supporting the conclusions of this article will be made available by the authors, without undue reservation.

## Ethics Statement

The studies involving human participants were reviewed and approved by King’s College London. The patients/participants provided their written informed consent to participate in this study.

## Author Contributions

EW-P and GT took a lead on data collection analysis and write up, with DC as collaborator. All authors contributed to the article and approved the submitted version.

## Funding

The authors were funded by Wellcome Trust grant no. 200796 (awarded to DC) and supported by the Oxford Health NIHR Biomedical Research Centre and NIHR Senior Fellowships (DC). The views expressed are those of the authors and not necessarily those of the NHS, the NIHR or the Department of Health.

## Conflict of Interest

The authors declare that the research was conducted in the absence of any commercial or financial relationships that could be construed as a potential conflict of interest.

## Publisher’s Note

All claims expressed in this article are solely those of the authors and do not necessarily represent those of their affiliated organizations, or those of the publisher, the editors and the reviewers. Any product that may be evaluated in this article, or claim that may be made by its manufacturer, is not guaranteed or endorsed by the publisher.

## Appendix 1

Social Cognitions about Face Masks Questionnaire (SCFM).

Please rate how much you believe the following statements from 0 to 100% (0%=not at all, 100%=completely).

**Table:**

If I wear a face mask/covering in social situations I believe…	0–100%
I’m keeping people safe	
I will look awkward	
I will appear confident	
People will stare at me	
People will think I’m rude if I speak with it on	
People will judge me negatively	
I will appear sweaty	
I will appear red	
I will look anxious	
I will look foolish	
I will look competent	
I will look stupid	
People will not understand what I say	
People will think I’m uncool	
People will think I’m boring	

## References

[ref1] Becker M. H. (1974). The health belief model and personal health behavior. Health Educ. Monogr. 2, 324–508.

[ref2] Connor K. Kobak K. Churhill L. Katzelnick D. Davidson J. (2001). Mini-SPIN: A brief screening assessment for generalized social anxiety disorder. Depress. Anxiety 14, 137–140. doi: 10.1002/da.1055 11668666

[ref3] Feng S. Shen C. Xia N. Song W. Fan M. Cowling B. J. (2020). Rational use of face masks in the COVID-19 pandemic. Lancet Respir. Med. 8, 434–436. doi: 10.1016/S2213-2600(20)30134-X, PMID: 32203710 PMC7118603

[ref4] Imperial College London (2020). How are behaviours changing in response to COVID-19?. Available at: http://www.coviddatahub.com. (Accessed August 24, 2020)

[ref5] Kroenke K. Spitzer R. L. Williams J. B. (2003). The patient health Questionnaire-2: validity of a two-item depression screener. Med. Care 41, 1284–1292. doi: 10.1097/01.MLR.0000093487.78664.3C, PMID: 14583691

[ref6] Nakayachi K. Ozaki T. Yukihide S. Yokoi R. (2020). Why do Japanese people use masks Against COVID-19, even Though masks are unlikely to offer protection From infection? Front. Psychol. 1:1918. doi: 10.3389/fpsyg.2020.01918 PMC7417658 32849127

[ref7] Rosenstock I. M. (1974). The health belief model and preventive health behavior. Health Educ. Monogr. 2, 354–386. doi: 10.1177/109019817400200405

[ref8] Seale H. Mak J. P. I. Razee H. MacIntyre R. C. (2012). Examining the knowledge, attitudes and practices of domestic and international university students towards seasonal and pandemic influenza. BMC Public Health 12:307. doi: 10.1186/1471-2458-12-307 22537252 PMC3447694

[ref9] Seeley-Wait E. Abbott M. J. Rapee R. M. (2009). Psychometric properties of the mini-social phobia inventory. Primary Care Companion J. Clin.l Psychiatry. 11, 231–236. doi: 10.4088/PCC.07m00576, PMID: 19956461 PMC2781035

[ref10] Sim S. W. Moey K. S. P. Tan N. C. (2014). The use of facemasks to prevent respiratory infection: a literature review in the context of the health belief model. Singap. Med. J. 55, 160–167. doi: 10.11622/smedj.2014037, PMID: 24664384 PMC4293989

[ref11] The Royal Society (2020) Face masks and coverings for the general public: Behavioural knowledge, effectiveness of cloth coverings and public messaging. Available at: https://royalsociety.org/-/media/policy/projects/set-c/set-c-facemasks.pdf?la=en-GBandhash=A22A87CB28F7D6AD9BD93BBCBFC2BB24. (Accessed August 25, 2020).

[ref12] van der Westhuizen H. Kotze K. Tonkin-Crine S. Gobat N. Greenhalgh T. (2020). Face coverings for covid-19: from medical intervention to social practice. BMJ 370:m3021. doi: 10.1136/bmj.m3021 32816815

[ref13] You Gov/Imperial College London (2020) COVID-19 Britons still won’t wear face masks. Available at: https://yougov.co.uk/topics/health/articles-reports/2020/06/04/covid-19-britons-still-wont-wear-face-masks. (Accessed August 25, 2020).

[ref14] Zhang C. Q. Chung P. K. Liu J. D. Chan D. K. C. Hagger M. S. Hamilton K. (2019). Health beliefs of wearing facemasks for influenza A/H1N1 prevention: A qualitative investigation of Hong Kong older adults. Asia Pac. J. Public Health 31, 246–256. doi: 10.1177/1010539519844082, PMID: 31007032

