# Supplementary material for: Belief in Protecting Others and Social Perceptions of Face Mask Wearing Were Associated With Frequent Mask Use in the Early Stages of the COVID Pandemic in the UK
Source: Front Psychol. 2021 Oct 22;12:680552. doi: 10.3389/fpsyg.2021.680552 (PMC8569128; doi:10.3389/fpsyg.2021.680552)
Supplement: Supplementary file 1 [file Table_1.pdf]

Supplementary material

Table shows guidance on Face Mask wearing across the UK both at the time the survey opened and any changes that took place to guidance between 7-24th July.

	**Guidance on wearing face masks in the UK at the time of completing the survey (7-24th July 2020)**	**New guidance announced during period survey open (7-24th July)**
England	Wearing mandatory on public transport and when visiting a hospital (since 15th June 2020). Recommended wearing in enclosed public spaces when social distancing not possible and when coming into contact with people you do not usually meet	Public told on 14th July that face masks would become mandatory in shops from the 24th July.
Wales	No mandatory wearing. Recommended to be worn in confined indoor spaces such as on public transport and shops but only if overcrowded.	Public told on 15th July that face masks would become mandatory on public transport from July 27th.
Northern Ireland	No mandatory wearing. Recommended in enclosed spaces where social distancing is not possible. Public were made aware on 2nd July that from July 10th wearing on public transport would become mandatory.	From July 10th wearing became Mandatory on public transport
Scotland	Face masks mandatory on public transport and on public transport premises (since 22 June 2020). Recommended in enclosed spaces, where physical distancing is more difficult and when in close contact with multiple people who are not members of your household, you should wear a face covering.<br><br>Public were aware on July 2nd than from July 10th wearing in shops would become mandatory.	From July 10 Face masks mandatory in shops
